# Superior Mesenteric Artery Syndrome: A Case Report of Two Surgical Options, Duodenal Derotation and Duodenojejunostomy

**DOI:** 10.1155/2016/8301025

**Published:** 2016-12-22

**Authors:** Yagan Pillay

**Affiliations:** Victoria Hospital, Prince Albert Parkland Health Region, 1521 6th Avenue West, Prince Albert, SK, Canada S6V 5K1

## Abstract

Superior mesenteric artery (SMA) syndrome is a rare cause of duodenal obstruction and its management is usually conservative with nasojejunal feeding. The pathophysiology entails the loss of the fat pad between the superior mesenteric artery and the abdominal aorta. This reduces the angle between the two vessels to less than 20 degrees with the resultant compression of the third part of the duodenum. The surgical management is usually a laparoscopic duodenojejunostomy. The two cases in our series had two different surgical procedures with good outcomes in both patients. The surgical management of each patient should be determined on its own merits irrespective of the standard of care.

## 1. Introduction

The surgical management of superior mesenteric artery (SMA) syndrome in the modern era is reserved for medically refractive cases.

Nasojejunal feeding is usually the first line of therapy and thought to offer a more durable alternative [1] without the attendant surgical morbidity.

When surgery is required, a laparoscopic duodenojejunal anastomosis is now the standard of care. Its success rate over a 5-year follow-up period is over 90 percent [2].

We present two cases of SMA syndrome with differing surgical approaches both of which have been successful. Strong's procedure in the first case has been followed up for over four years with no attendant morbidity and a complete resolution of symptoms.

In the second case we were unable to perform Strong's procedure due to a previous subtotal colectomy performed on the patient for colitis. She had a duodenojejunostomy. Her follow-up over the past two years has been uneventful.

## 2. Case Report One

A 45-year-old Caucasian female presented with abdominal pain over the past two years and a concomitant sixty-pound weight loss that was unintentional.

The pain was in the epigastrium.

She had no change in her bowel habits but she did have postprandial vomiting about 30 minutes after each meal. The vomitus was bile stained.

There was no food pain association.

She drank occasionally and was a regular smoker. She had no other significant medical or surgical history and no known allergies.

Her physical examination was normal. She had been examined by a number of physicians in the past with no significant clinical findings.

Her blood results and an abdominal ultrasound were normal.

A subsequent gastroscope was also normal.

**Figure 1:**
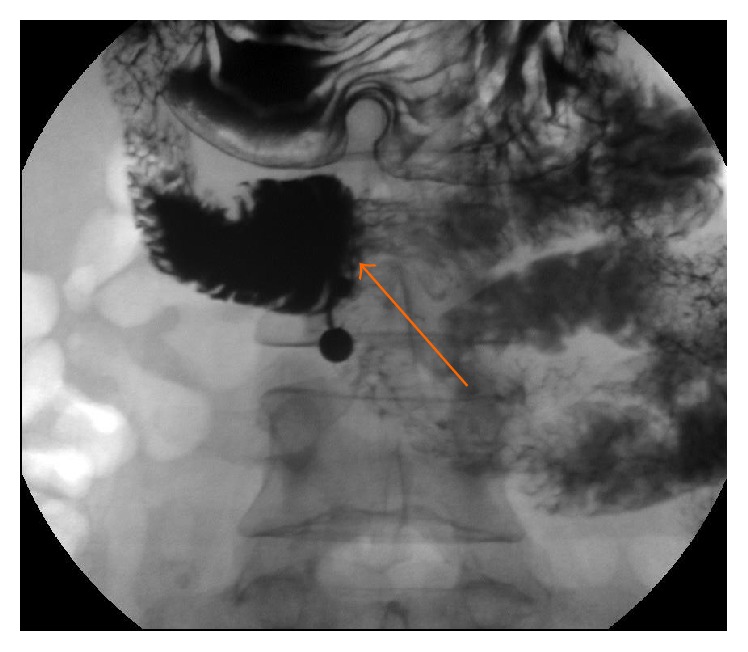
Duodenal compression of the third part (orange arrow) with delay in contrast passage (Case Report One).

Further radiological imaging with a barium meal and follow through showed a holdup of contrast in the third part of the duodenum (Figure 1).

**Figure 2:**
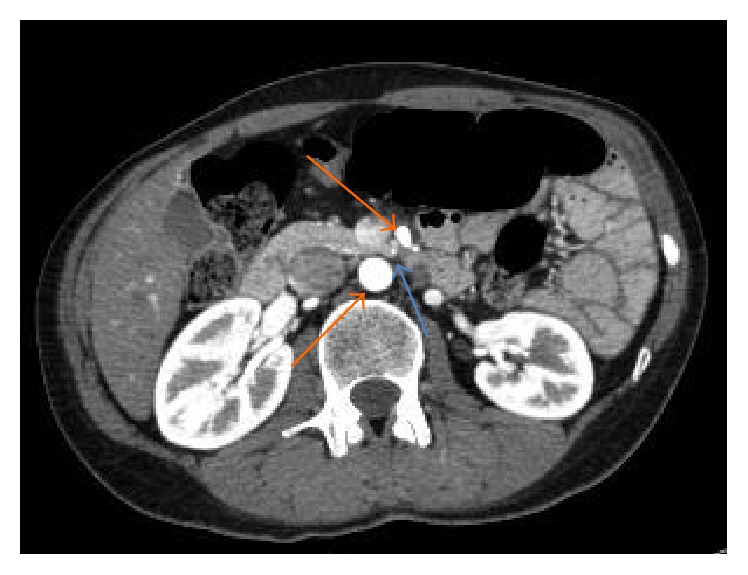
CT scan (axial view) with duodenal compression (blue arrow) between the aorta and superior mesenteric artery (orange arrows) (Case Report One).

**Figure 3:**
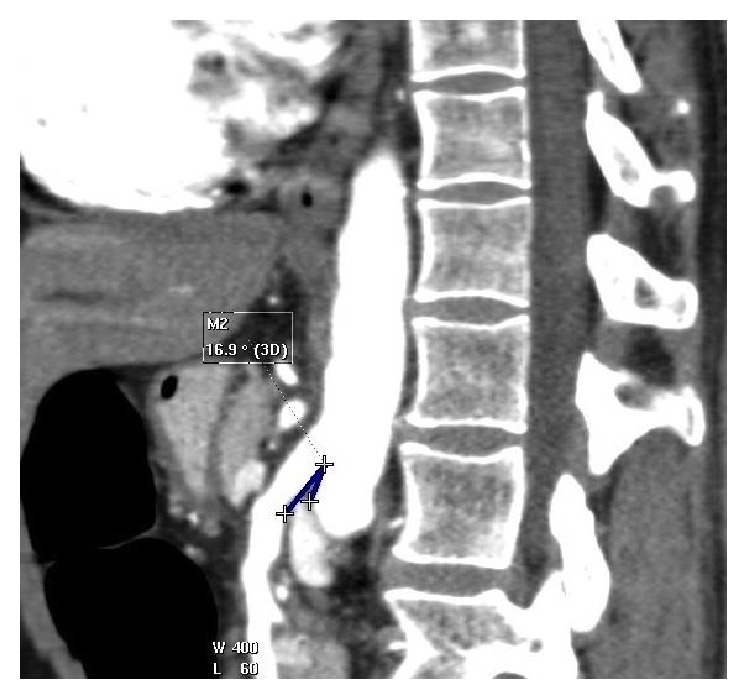
CT sagittal view showing angle of 16.9 degrees between the superior mesenteric artery and aorta (Case Report One).

A subsequent Computerised Tomography (CT) scan showed an acute angle (16.9 degrees) between the superior mesenteric artery and the aorta which confirmed the diagnosis (Figures 2 and 3).

After an extensive discussion with the patient and her family, she opted for surgery rather than conservative management. She was unwilling to try nasojejunal feeds or total parenteral nutrition (TPN) in the interim.

She remained adamant about surgery even after referral to a second surgeon.

Due to the surgeon's lack of experience with laparoscopic duodenojejunostomy as well as a laparoscopic Strong's procedure, the patient was offered an open exploratory laparotomy.

**Figure 4:**
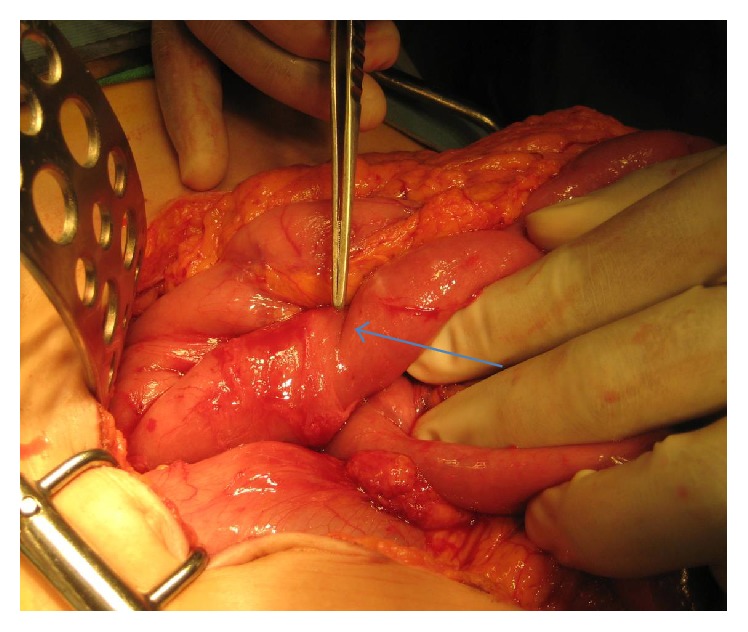
Superior mesenteric artery indentation on the third part of duodenum (blue arrow) (Case Report One).

Intraoperatively we found duodenal compression of the third part due to the superior mesenteric artery (Figure 4). She also had lymph nodes around the third part of the duodenum so the decision was made intraoperatively to perform Strong's procedure and not a duodenojejunostomy. An excisional biopsy of one lymph node was carried out as well.

The pathology of the lymph node confirmed a sinus histiocytosis and she was referred to a haematologist. The haematologist elected to manage the histiocytosis conservatively due to the self-limiting nature of the disease.

The patient made an uneventful recovery. Postoperatively she recovered 30 pounds of weight over a six-month period. She was also able to tolerate full meals without any postprandial vomiting.

At four-year follow-up, her symptoms have abated and her only surgical comorbidity was an incisional hernia that was repaired with a mesh two years later.

## 3. Case Report Two

A 44-year-old Caucasian female presented with abdominal pain, weight loss, nausea, and vomiting. She also suffered from chronic diarrhea. This had been ongoing for two years following a subtotal colectomy and ileosigmoid anastomosis.

The subtotal colectomy was for clostridium difficile colitis not responsive to antibiotics.

She also used medical marijuana for pain control due to an opiate allergy.

Medical management of her diarrhea was ineffective.

Her medical history included depression and migraine headaches which were both well controlled with medication.

Her CT scan showed duodenal compression of the third part as well as an acute angle (14.7 degrees) between the superior mesenteric artery and the abdominal aorta (Figures 8 and 9). The diagnosis of superior mesenteric artery syndrome was entertained and confirmed by a barium meal and follow through (Figure 7).

After a discussion with the patient, an informed consent was obtained for an exploratory laparotomy. Nonoperative management was not contemplated given her long standing issues with diarrhea as a result of her initial surgery for colitis.

Intraoperatively she had an obvious compression of the third part of the duodenum (Figure 10). Initially after mobilization of the duodenojejunal flexure an attempt was made at duodenal derotation (Strong's procedure). Intraoperatively this resulted in bowel ischaemia due to malrotation of her mesentery as a result of a previous ileosigmoid anastomosis following a subtotal colectomy. Strong's procedure was abandoned and a Roux-en-Y duodenojejunostomy was performed (Figure 11).

Her recovery was uneventful and she was discharged home.

Her two-year follow-up showed complete resolution of her upper GI symptoms.

## 4. Discussion

Superior mesenteric artery (SMA) syndrome is a rare cause of small bowel obstruction [4]. The diagnosis is confirmed by the loss of an angle between the superior mesenteric artery and the abdominal aorta to less than 20 degrees [5].

The distance between the two vessels is also less than 6 mm (the normal distance is 8–12 mm) [5].

**Figure 5:**
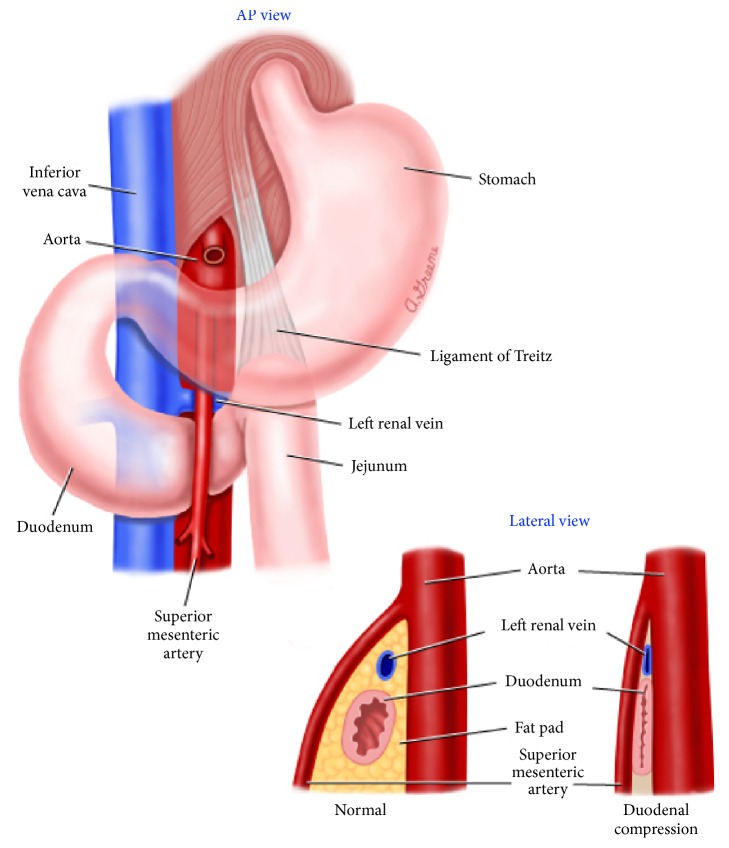
Superior mesenteric artery (SMA) syndrome. The superior mesenteric artery arises from the anterior aspect of the aorta at the level of the L1 vertebral body. It is enveloped in fatty and lymphatic tissue and extends in a caudal direction at an acute angle into the mesentery. In the majority of patients, the normal angle between the superior mesenteric artery and the aorta is between 38 and 65 degrees. Superior mesenteric artery syndrome is characterized by compression of the third portion of the duodenum due to narrowing of the space between the superior mesenteric artery and aorta and is primarily attributed to loss of the intervening mesenteric fat pad. With superior mesenteric artery syndrome, the angle between the superior mesenteric artery and the aorta can be narrowed to as little as 6 degrees (Case Report One) (see [3]).

It is this loss of obliquity that results in the compression of the third part of the duodenum as it runs between the superior mesenteric artery and the aorta from right to left [6] (Figure 5).

While medical treatment remains the popular approach there has not been enough follow-up of the surgical management [1]. Surgery is usually contemplated if there is a failure of conservative management.

**Figure 6:**
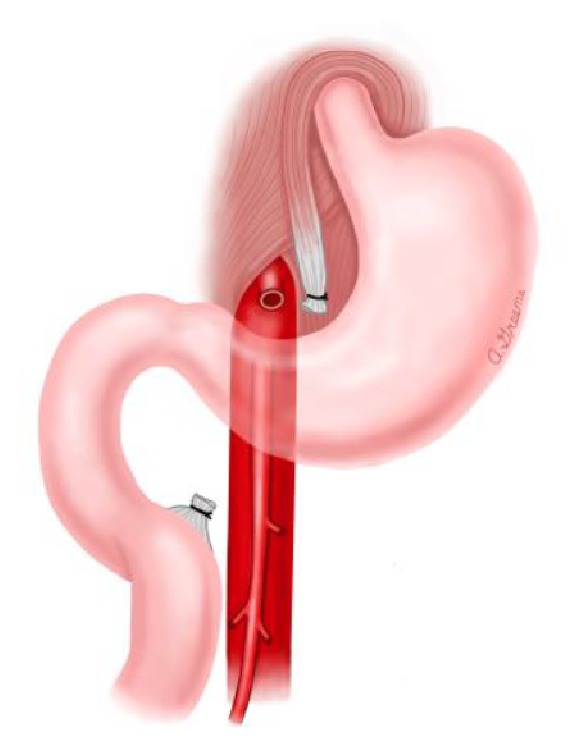
Strong's procedure. Strong's procedure mobilizes the duodenum by dividing the ligament of Treitz. Once the duodenal-jejunal junction is mobilized, the duodenum is positioned to the right of the superior mesenteric artery (Case Report One) (see [3]).

**Figure 7:**
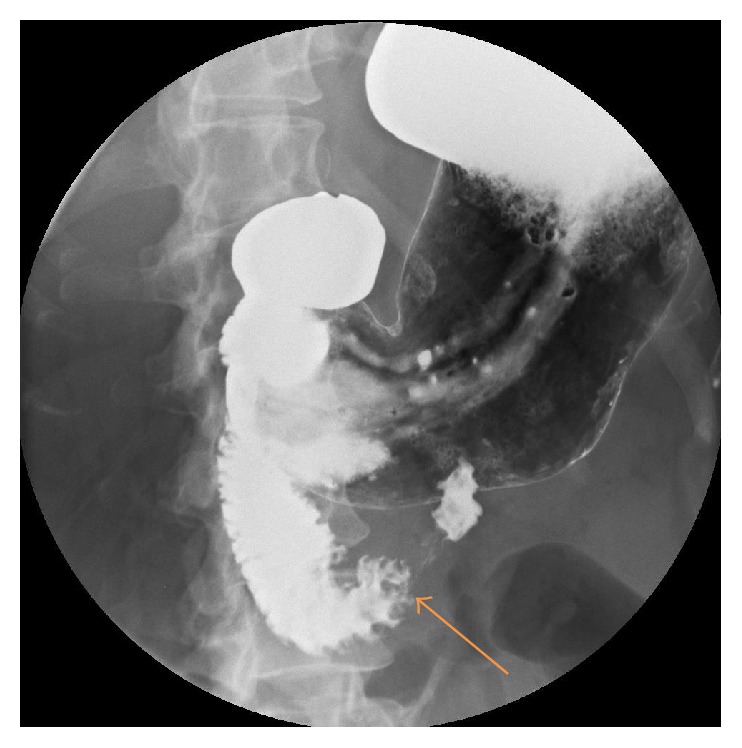
Duodenal compression of the third part (orange arrow) on barium meal and follow through (Case Report Two).

**Figure 8:**
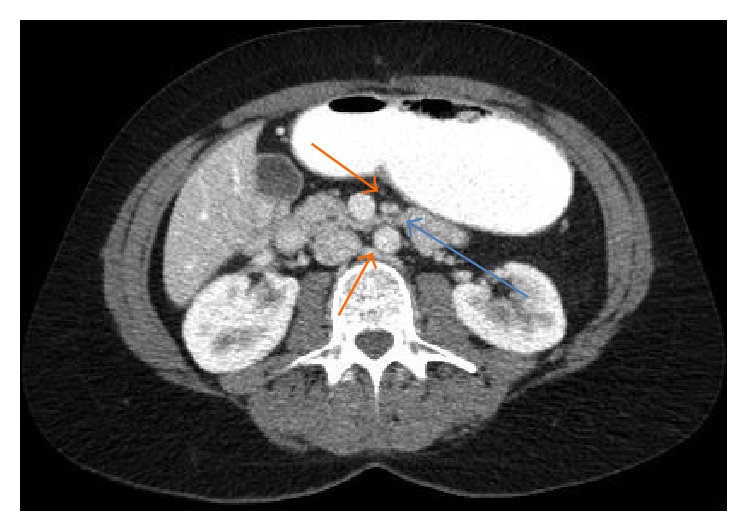
Duodenal compression on CT scan (blue arrow) between the aorta and superior mesenteric artery (orange arrows) (Case Report Two).

**Figure 9:**
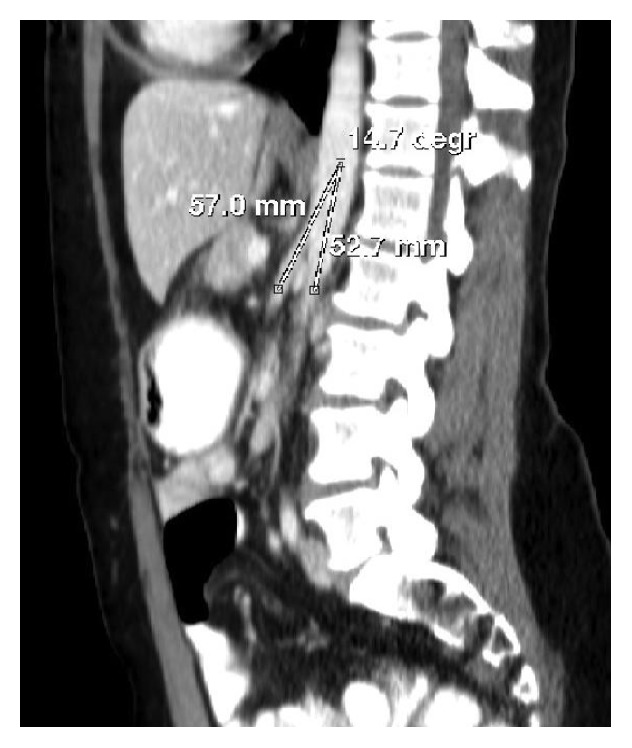
CT sagittal view showing an angle of 14.7 degrees between the SMA and aorta (Case Report Two).

**Figure 10:**
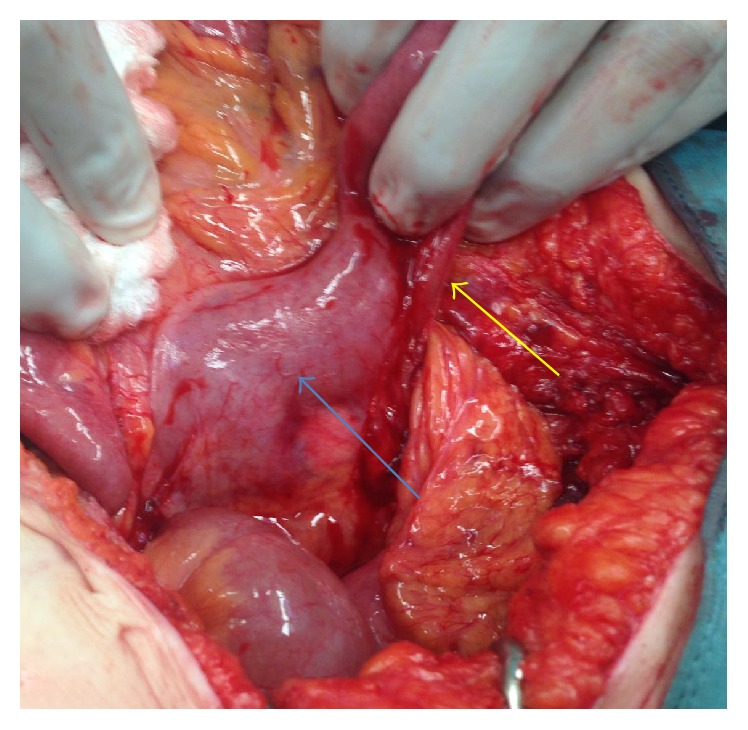
Duodenal compression of the third part (blue arrow) and DJ flexure mobilized (yellow arrow) (Case Report Two).

**Figure 11:**
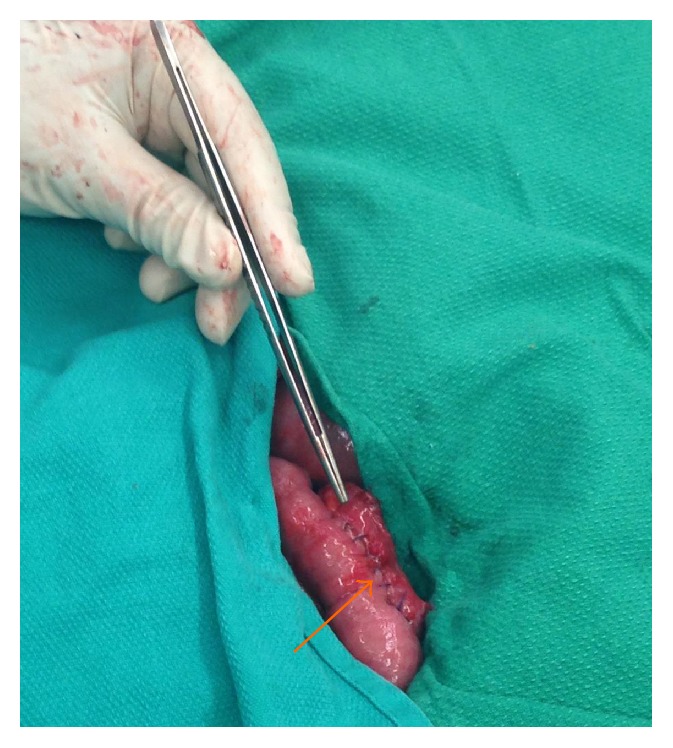
Completed duodenojejunostomy (jejunum, orange arrow) (Case Report Two).

Historically the surgical management consisted of three procedures, namely, Strong's procedure (Figure 6), duodenojejunostomy, or a gastrojejunostomy [7].

The gastrojejunostomy is usually undertaken in the presence of gastric distention which has caused gastroparesis and delayed emptying of the stomach.

The laparoscopic duodenojejunostomy is now the surgical procedure of choice with success rates of over 90 percent over the long term [2].

Strong's procedure has a failure rate of over 25 percent and is not currently recommended. Its durability over the long term however has been well demonstrated [8].

Vascular implantation of the superior mesenteric artery is only used as a last resort due to its attendant morbidity.

In the first case a duodenojejunostomy could not be performed as a result of the presence of lymph nodes around the duodenojejunal (DJ) flexure. Intraoperatively the aetiology of the lymphadenopathy could not be ascertained so the decision was made not to perform a surgical anastomosis.

Strong's procedure (Figure 6) encompasses a derotation of the embryonal rotation of the small bowel [9]. This surgical malrotation results in the duodenum being positioned to the right of the patient's midline once the DJ flexure is mobilized.

This then places it lateral to the oblique angle between the superior mesenteric artery and the abdominal aorta.

In the second case, the patient's previous surgery prevented Strong's procedure as the duodenal malrotation caused a bowel obstruction due to inadvertent twisting at the previous ileosigmoid anastomosis. This caused an ischaemia due to occlusion of the mesenteric blood supply.

A Roux-en-Y duodenojejunostomy was performed instead.

The two cases demonstrate clearly that there is no standard approach to this pathology and that each patient needs to be assessed on their own merit [10].

There is no “one size fits all” policy. Strong's procedure while it is no longer the standard of care is still a viable surgical alternative with demonstrable long-term outcomes [8]. Surgical management should only proceed after a definite diagnosis has been obtained. Dedicated CT imaging with a barium meal and follow through is of utmost importance in showing the duodenal occlusion in the third part. There needs to be an extensive discussion with the patient and her family as to the surgical options as no one surgical option is the answer and there may be a change in the surgery performed as shown in the second case.

## References

[1] Chan D. K., Mak K. S., Cheah Y. L. (2012). Successful nutritional therapy for superior mesenteric artery syndrome. *Singapore Medical Journal*.

[2] Mandarry M. T., Zhao L., Zhang C., Wei Z. Q. (2010). A comprehensive review of superior mesenteric artery syndrome. *European Surgery*.

[3] Scovell S., Hamdan A. (2015). Superior mesenteric artery syndrome. *UpToDate, Post, Ted*.

[4] Valdes A., Cárdenas O., Espinosa A., Villazón O., Valdes V. (2005). Superior mesenteric artery syndrome. *Journal of the American College of Surgeons*.

[5] Derrick J. R., Fadhli H. A. (1965). Surgical anatomy of the superior mesenteric artery. *The American Surgeon*.

[6] Gustafsson L., Falk A., Lukes P. J., Gamklou R. (1984). Diagnosis and treatment of superior mesenteric artery syndrome. *British Journal of Surgery*.

[7] Pourhassan S., Grotemeyer D., Fürst G., Rudolph J., Sandmann W. (2008). Infrarenal transposition of the superior mesenteric artery: a new approach in the surgical therapy for Wilkie syndrome. *Journal of Vascular Surgery*.

[8] Ha C. D., Alvear D. T., Leber D. C. (2008). Duodenal derotation as an effective treatment of superior mesenteric artery syndrome: a thirty-three year experience. *American Surgeon*.

[9] Merrett N. D., Wilson R. B., Cosman P., Biankin A. V. (2009). Superior mesenteric artery syndrome: diagnosis and treatment strategies. *Journal of Gastrointestinal Surgery*.

[10] Yakan S., Calıskan C., Kaplan H., Deneclı A. G., Coker A. (2013). Superior mesenteric artery syndrome: a rare cause of intestinal obstruction. Diagnosis and surgical management. *Indian Journal of Surgery*.

